# California of To-Day

**Published:** 1866-11

**Authors:** 


### CALIFORNIA OF TO-DAY.

BY H. L. W.

You ask me to give you upon paper some of the little incidents was relating to you last night. Here is one of our California ranches, very much at your service.

You understand, cousin, that Petaluma is the central one of the three rich agricultural valleys—Napa, Petaluma, and Sonora—which are so often referred to in accounts of California. You are also familiar with the oft-repeated descriptions of their fertility and beauty; I shall not therefore expatiate upon that theme, but proceed to the little visit that interested you.

It was the noon of a warm and bright Summer's day that we drove up to the gates of General H——'s ranche, and into the vineyard, through which a carriage-drive wound up to the dwelling. About midway this drive we were brought to a halt for a few minutes, by a picturesque and to me novel spectacle. Three large wagons intercepted the way, laden to the brim, literally overflowing with ripe grapes of the finest quality, odor and color, just gathered for the wine-press.

Several men were at work among the vines, each with a bushel-basket for the reception of the grapes when gathered. These were speedily filled with huge, luscious clusters, of the most beautiful coloring, from a transparent ruby to the most royal purple. One after another the men came up with these baskets poised upon the shoulder, and deposited them in the wagons. The last load was nearly completed when we entered. They soon drove on and made way for us.

The house was old and oddly-fashioned, but improvements were going on of additional apartments spacious and commodious. The site was very fine, standing upon elevated ground, backed by high and picturesque hills, and commanding from the veranda in front a varied and extensive prospect of many miles—hill, dale, and woodland, just flushed with the mellow tints of departing Summer; and far off to the left, just melting in the distance of enchantment, the fair blue waters of the Pacific.

I remember it well—that sweetly-tinted picture; it held my charmed eyes for some minutes while we stood upon the veranda waiting for admission. We entered to look upon pictures of a totally different character. The aspect of the room surprised me; was I in reality on a California *ranche?* was this the farm-house of a wild new country?

We were in the chief apartment of the dwelling. The room seemed to me of good size and height; I could not give the dimensions in feet and inches, but it was rather large, and the walls all round from floor to ceiling were covered with costly paintings from the Old World. These, I believe, had been brought from Europe by General H——.

Pictures of every description, old family portraits, vaguely hinting at their own histories; landscapes, miniatures, and fancy pieces of all forms and sizes, richly framed, and making the old house gorgeous and glowing with beauty. The room was crowded with the costly furniture of an elegant drawing-room; there was a fine piano, and more pretty *bijouterie* than I could recount.

I was very much interested in Mrs. H——, who received and entertained us most courteously. She is from Hungary, a handsome and elegant woman, of medium height, and fine form, and so youthful in appearance, with her gay, vivacious manner, that it was difficult to credit that the several portraits of grown sons and half-grown daughters upon the walls were representations of her children.

She came in fresh from the pleasant occupation of preserving, and though neatly attired in a handsome French chintz, buckled at the waist with gold, apologized for her attire.

"Why, Madame," said I, "I should suppose you had no occasion for anything better than a chintz in the country, particularly *this* country." To which she replied, "That at this season she found it more necessary to be *dressed*, on the ranche, than in San Francisco, owing to the reception of numerous visitors. Moreover," she added, with a playful grace peculiar to her manner, "there are our husbands; if we fail to make ourselves charming to them, we must not complain if we find ourselves neglected. May be," she said, laughing, "they'll be hunting up new sweet-hearts! No!" shaking her head, "that will never do!" She liked ranche-life, her occupation was constant and agreeable; it was something new to her, too. She conducted us over the place, explaining and exhibiting everything that was likely to interest us; through wide and well-made walks, fringed on either hand with fruit trees or shrubbery, and plucking as she went ripe figs and clusters of purple grapes, with which she filled our hands.

We first went to the wine-cellar. A grotto of fifty feet in extent had been excavated out of the solid rock in the side of a hill. This was filled with barrels of new wine. The front part of this formed quite a large apartment. Here were the wine-presses, and two or three men were at work making and barrelling wine. A pleasant occupation enough, I should think.

From this we went to look at the lady's bath-house, which was built over a warm spring. Nature had certainly done her part towards making this place an agreeable residence, even to tempering the bath of a dainty lady. In another part of the grounds was the General's study—a small building, of one room, hidden away in a sweet seclusion beneath fine old trees, which sheltered it beneath their protecting arms from dust, rain, and sun. We did not enter this sanctum, as it was locked, but through the glass of the long French windows we looked in upon a handsomely-furnished room, fitted up like a library, shelves from floor to ceiling filled with books, upon the floor a gay carpet, a table with writing implements and papers, an easy morocco-cushioned chair; and through the glass doors on either hand charming glimpses of green foliage and flowering shrubs.

This little snuggery delighted me. To reach it, we crossed a fine brook, the sweet lulling sound of whose gurgling waters was distinctly heard here blending with the songs of the many birds overhead.

I believe I have written this very nearly as I related it to you. It is now nearly two years since I was there, and within this time the wealth and taste of the proprietors have doubtless effected great improvements.

There were several other ranches, or farms, within this valley worthy of a record, but this will suffice for this article.

